# Regulation of *mtl *operon promoter of *Bacillus subtilis*: requirements of its use in expression vectors

**DOI:** 10.1186/1475-2859-10-83

**Published:** 2011-10-20

**Authors:** Kambiz Morabbi Heravi, Marian Wenzel, Josef Altenbuchner

**Affiliations:** 1 Institut für Industrielle Genetik, Universität Stuttgart, Allmandring 31, 70569 Stuttgart, Germany

**Keywords:** PTS, PRD, Activator, Catabolite repression, Mannitol operon

## Abstract

**Background:**

Several vector systems have been developed to express any gene desired to be studied in *Bacillus subtilis*. Among them, the transcriptionally regulated promoters involved in carbohydrate utilization are a research priority. Expression systems based on *Bacillus *promoters for xylose, maltose, and mannose utilization, as well as on the heterologous *E. coli *lactose promoter, have been successfully constructed. The promoter of the *mtlAFD *operon for utilization of mannitol is another promising candidate for its use in expression vectors. In this study, we investigated the regulation of the *mtl *genes in order to identify the elements needed to construct a strong mannitol inducible expression system in *B. subtilis*.

**Results:**

Regulation of the promoters of *mtlAFD *operon (*P_mtlA_*) and *mtlR *(*P_mtlR_*) encoding the activator were investigated by fusion to *lacZ*. Identification of the *P_mtlA _*and *P_mtlR _*transcription start sites revealed the σ^A ^like promoter structures. Also, the operator of *P_mtlA _*was determined by shortening, nucleotide exchange, and alignment of *P_mtlA _*and *P_mtlR _*operator regions. Deletion of the mannitol-specific PTS genes (*mtlAF*) resulted in *P_mtlA _*constitutive expression demonstrating the inhibitory effect of EIICB^Mtl ^and EIIA^Mtl ^on MtlR in the absence of mannitol. Disruption of *mtlD *made the cells sensitive to mannitol and glucitol. Both *P_mtlA _*and *P_mtlR _*were influenced by carbon catabolite repression (CCR). However, a CcpA deficient mutant showed only a slight reduction in *P_mtlR _*catabolite repression. Similarly, using *P_groE _*as a constitutive promoter, putative *cre *sites of *P_mtlA _*and *P_mtlR _*slightly reduced the promoter activity in the presence of glucose. In contrast, glucose repression of *P_mtlA _*and *P_mtlR _*was completely abolished in a Δ*ptsG *mutant and significantly reduced in a MtlR (H342D) mutant.

**Conclusions:**

The *mtl *operon promoter (*P_mtlA_*) is a strong promoter that reached a maximum of 13,000 Miller units with *lacZ *as a reporter on low copy plasmids. It is tightly regulated by just one copy of the *mtlR *gene on the chromosome and subject to CCR. CCR can be switched off by mutations in MtlR and the glucose transporter. These properties and the low costs of the inducers, i.e. mannitol and glucitol, make the promoter ideal for designing regulated expression systems.

## Background

For many years, *Bacillus subtilis *has been vastly investigated not only as the model of Gram-positive bacterium, but also as an expression platform for homologous and heterologous gene expression. Efficient secretion of enzymes such as proteases, lipases, and amylases along with its safe use and completely sequenced genome is promising for industrial applications. However, several bottlenecks exist that limit the expression potential, e.g. low level of heterologous protein production, instability of the plasmids, inclusion body formation, and production of extracellular proteases [1]. So far, several expression systems have been developed in order to overcome the low production categorized to inducer-specific promoters, growth-phase promoters and autoinducible promoters [2]. Among them, systems based on *P_spac _*[3] and *P_xyl _*[4] have been widely used. Recently, two other systems were developed based on *P_manP _*[5] and *P_glv _*[6] promoters.

*B. subtilis *utilizes many carbohydrates via the phosphoenolpyruvate-dependent phosphotransferase system (PTS). In a PTS pathway, the sugar is translocated across the cytoplasmic membrane and concomitantly phosphorylated by a sugar-specific permease, i.e. Enzyme II (EII). The phosphate is provided by phosphoenolpyruvate (PEP), which is synthesized during glycolysis. The phosphate transfer from PEP to sugar is catalyzed by PTS Enzyme I, phosphocarrier protein HPr, and EIICBA. The latter is responsible for the uptake of the sugars. In addition to uptake and phosphorylation, the PTS is involved in the regulation of catabolic operons. Hereby, the PTS ensures the hierarchical utilization of carbon sources, a phenomenon known as carbon catabolite repression (CCR) [7].

The CCR of sugar operons is dependent on phosphorylated intermediates of the PTS. Despite the fact that the phosphate transfer cascade is similar in both Gram-positive and Gram-negative bacteria, there are some outstanding differences in the regulation of CCR. While in Gram-negative bacteria, such as *Escherichia coli*, the phosphorylation status of the cytoplasmic EIIA domain for glucose (EIIA^Glc^) plays the main role, it is HPr in low G+C Gram-positive bacteria, such as *B. subtilis*, that is dominant. In *B. subtilis*, HPr shows two phosphorylation sites. Phosphorylation at His15 is needed for the transfer reaction to EIIA component, whereas the phosphorylation at Ser46 plays a regulatory role [8]. HPr (Ser46~P) (or Crh (Ser46), which shares 45% identity in amino acid sequence with HPr, but contains only the serine regulatory site) is able to interact with catabolite control protein A (CcpA) in order to form a complex. Afterwards, this complex binds to so-called *cre *(catabolite-responsive element) sites in the promoter region of the relevant genes and leads to positive or negative regulation of the genes depending on the location of *cre *(for review see [9-14]). In addition to CcpA-dependent carbon catabolite repression, there are also CcpA-independent systems in *B. subtilis *[15,16]. For instance, trehalose and sucrose are known to be regulated by the inducer exclusion mechanism. In these systems the transporter lacks the specific EIIA domains. Therefore, the phosphate is transferred from the EIIA domain of EIICBA^Glc ^to the EIIB domains of the trehalose and sucrose transporters. Glucose competes with the phosphorylation of the EIIB domains and hereby reduces sucrose and trehalose transport [17,18]. A second, more important CcpA independent CCR occurs via PTS regulation domains (PRDs) in transcriptional antiterminators and activators. PRD containing antiterminators have two PRDs in addition to a RNA binding site. One PRD (PRDII) is phosphorylated by HPr (His15) and the other (PRDI) is phosphorylated by the specific PTS transport system, which is regulated by the antiterminator. For antiterminator activity, usually the PRDI has to be dephosphorylated and the PRDII phosphorylated, which is the case in the presence of the specific sugar and absence of glucose. PRD containing activators are similar, but contain a DNA binding site instead of the RNA binding site, and they have additional domains like the mannose activator, which contains an EIIA and EIIB domain like the PTS transporters [16,19-22].

**Figure 1:**
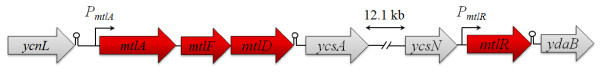
**Genetic map of *mtl *operon**. Organization of the mannitol PTS encoding genes consisting of *mtlAFD *and its regulator *mtlR *in *B. subtilis *168 is depicted.

Mannitol consumption by *B. subtilis *is another example for regulation by PRD containing activators. The polyol is taken up by the mannitol system as one of the 17 PTS in *B. subtilis*, which were identified by genome sequencing and protein annotations. Four genes are needed: *mtlA *(encoding enzyme IICB^Mtl^), *mtlF *(encoding enzyme IIA^Mtl^), *mtlD *(encoding mannitol-1-phosphate dehydrogenase), and *mtlR *(encoding the transcriptional activator) (Figure 1). The first genes comprise the *mtl *operon, whereas *mtlR *is located at 14.4 kb downstream from the *mtl *operon [23-27]. Previously, it was shown that transcription of the mannitol operon is activated by MtlR [28]. The MtlR protein consists of a DNA binding domain and two PRD domains as well as EIIA^Mtl^-like and EIIB^Gat^-like domains. Recent studies of MtlR have indicated that phosphorylation of the PRDII domain of MtlR by HPr (His15), as well as the absence of phosphorylation of EIIA^Mtl^-like and EIIB^Gat^-like domains, respectively, have a stimulatory effect on the activity, whereas the dephosphorylation of PRDI reduced the MtlR activity 5 to 25% [29]. Besides mannitol, the transcription of the *mtl *operon is induced by glucitol, a non-PTS sugar. Also, *mtlD *is required for the assimilation of glucitol in *B. subtilis *[28]. Basically, glucitol is taken up by a H^+^-symporter (encoded by *gutP*) without any chemical modification and oxidized to fructose by glucitol dehydrogenase (*gutB*). In addition to the GutP transporter, it was observed that glucitol could be weakly taken up by EIICBA^Mtl ^and phosphorylated to glucitol 6-phosphate [30].

In this study, we characterize the transcriptional activity of both *P_mtlA _*and *P_mtlR _*depending on the presence of mannitol and other sugars as well as the influence of various mutations on the regulation of the two mannitol promoters.

## Methods

### Strains, media and growth condition

Bacterial strains used in this study are listed in Table 1. Transformants of *E. coli *and *B. subtilis *were selected on LB agar [31], supplemented with ampicillin (100 μg ml^-1^) or spectinomycin (100 μg ml^-1^) depending on the plasmid antibiotic marker. Unless otherwise specified, knock-out *B. subtilis *mutants were selected on LB agar, containing erythromycin (5 μg ml^-1^) or chloramphenicol (5 μg ml^-1^). Mutations were confirmed by cultivating the mutants in modified Spizizen salts medium [32]. Trisodium citrate, glucose, and trace elements were replaced by 0.02% (w/v) casamino acids. For tryptophan auxotrophic *B. subtilis *168 and its derivatives, 50 μg ml^-1 ^tryptophan was added. The carbon source was either 1% (w/v) sterile-filtered mannitol, glucitol or glucose. All of the strains were incubated at 37^°^C under a shaking condition at 200 rpm.

**Table 1:** Strains and plasmids used in this study

Strain or plasmid	Genotype or relevant structure	Source, reference, or construction
*E. coli*		
JM109	*recA1, endA1, gyrA96, thi, hsdR17, supE44, relA, λ^-^*, Δ(*lac-proAB*), [*F', traD36, proAB, lacI^q^Z*Δ*M15*]	[69]
*B. subtilis*		
3NA	*spo0A3*	[70]
168	*trpC2*	Bacillus Genetic Stock Center
KM12	*spo0A3 *Δ*mtlAF::ermC*	3NA transformed with pKAM5
KM13	*spo0A3 *Δ*mtlAFD::ermC*	3NA transformed with pKAM6
KM15	*spo0A3 *Δ*mtlR::ermC*	3NA transformed with pKAM4
KM37	*spo0A3 mtlD::ermC*	3NA transformed with pKAM14
KM39	*spo0A3 *Δ*gutRBPydjE::cat*	3NA transformed with pKAM13
KM40	*spo0A3 *Δ*mtlAFD::ermC ΔgutRBPydjE::cat*	KM13 transformed with pKAM13
KM162	*spo0A3 *Δ*mtlR::ermC, hisI', spc*	KM15 transformed with pHM30
KM163	*spo0A3 mtlR-H342D*	KM162 transformed with pKAM66
MW312	*spo0A3 (ptsG-pstH')::ermC*	3NA transformed with pMW312.2
MW373	*spo0A3 *Δ*ptsG*	MW312 transformed with pMW373.3
TQ303	*spo0A3 *Δ*ccpA::ermC*	[5]
TQ432	*trpC2 ptsH-H15A amyE::cat*	[5]
Plasmids		
pHM30	*hisF, hisI', spc, yvcA, yvcB, bla*	[37]
pHM31	*hisF, hisI, yvcA, yvcB, bla*	[37]
pKAM1	*spc, ter- P_mtlA _*(-181/-1)^α ^*-lacZ-ter, repA*	This study
pKAM4	*'ycsN-ermC-ydaB', bla, spc*	This study
pKAM5	*'ycnL-ermC-P_mtlA_-mtlD', bla, spc*	This study
pKAM6	*'ycnL-ermC-ycsA', bla, spc*	This study
pKAM9	*spc, ter- P_mtlA _*(-161/-1) *-lacZ-ter, repA*	This study
pKAM12	*spc, ter- P_mtlA_' *(-161/-32)*-lacZ-ter, repA*	This study
pKAM13	*'ydjC-cat-pspA', bla, spc*	This study
pKAM14	*'mtlD::ermC, bla, spc*	This study
pKAM18	*spc, ter-P_mtlR_-lacZ-ter, repA*	This study
pKAM27	*spc, ter- P_mtlA _*(-161/-32)*[cttta→gaaat] *-lacZ-ter, repA*	This study
pKAM43	*spc, ter- *'*P_mtlA _*(-157/-32) *-lacZ-ter, repA*	This study
pKAM44	*spc, ter- P_mtlA_' *(-161/-52) *-lacZ-ter, repA*	This study
pKAM45	*spc, ter- P_mtlA _*(-161/-32)*[ctttc→gaaag] *-lacZ-ter, repA*	This study
pKAM48	*spc, ter- *'*P_mtlA _*(-159/-32) *-lacZ-ter, repA*	This study
pKAM49	*spc, ter- *'*P_mtlA _*(-155/-32) *-lacZ-ter, repA*	This study
pKAM52	*spc, ter- P_mtlA _*(-161/-32)*[ttcaa→aagtt] *-lacZ-ter, repA*	This study
pKAM57	*spc, ter- *'*P_mtlA _(-153/-32) -lacZ-ter, repA*	This study
pKAM58	*spc, ter- *'*P_mtlA _(-149/-32) -lacZ-ter, repA*	This study
pKAM59	*spc, ter- *'*P_mtlA _(-151/-32) -lacZ-ter, repA*	This study
pKAM66	*hisF, hisI, mtlR-H342-D, yvcA, yvcB, bla*	This study
pKAM84	*spc, ter- P_mtlA _*(-161/-32)*[gtcct→cagga] *-lacZ-ter, repA*	This study
pKAM88	*spc, ter- P_groE _- *(*cre_PmtlA_*) *-*UTR*_PmtlR_-lacZ-ter, repA*	This study
pKAM89	*spc, ter- P_groE _- *(*cre_PmtlR_*) *-*UTR*_PmtlR_-lacZ-ter, repA*	This study
pKAM90	*spc, ter- P_groE _-*(*cre_acsA_*) *-*UTR*_PmtlR_-lacZ-ter, repA*	This study
pKAM91	*spc, ter- P_groE _- *(*cre_mtlA_*) *-*UTR*_PmtlR_-lacZ-ter, repA*	This study
pKAM92	*spc, ter- P_mtlA _*(-161/-32)*[ccaaa→ggttt] *-lacZ-ter, repA*	This study
pKAM101	*spc, ter- P_groE _-*UTR*_PmtlR_-lacZ-ter, repA*	This study
pMW312.2	*glcT'-ermC-ptsHI', bla, spc*	This study
pMW363.1	*ter, spc, manP'-cat-yjdB-yjdA', bla*	Laboratory Stock
pMW373.3	*'glcT-P_ptsGHI_-ptsH', bla, spc*	This study
pSUN279.2	*ter-P_manR_-manR-P_manP_-lacZ-ter, repA, ori_pUC18_, ori_pBS72_, bla, spc*	[5]
pSUN338.3	*yvcL-ermC-yvcN, bla, spc*	[5]
pSUN356.7	*manR'-ermC-manA', bla, spc*	[5]

^α ^The numbers are showing the position of the sequence with respect to *mtlA *start codon.

For induction of the *mtl *promoters, 85 ml LB medium in 500 ml Erlenmeyer flasks were inoculated by an overnight culture in a dilution of 1:50 and incubated at 37^°^C under shaking condition (200 rpm). Aliquots of 8 ml with an OD_600 _of 0.4 were divided in a 100 ml Erlenmeyer flask and subsequently induced by addition of different carbohydrates to a final concentration of 0.2% (w/v) each, i.e. mannitol, mannitol + glucose, glucose, mannitol + xylose, xylose, glucitol, glucitol + glucose. Cultures were harvested 1 h after addition of sugars and used for enzyme activity studies. As a defined medium, Spizizen salts medium (SSM) (14 g of K_2_HPO_4_, 6 g of KH_2_PO_4_, 2 g of (NH_4_)_2_SO_4_, 1 g of trisodium citrate, 0.2 g of MgSO_4_.7H_2_O, and 10 g of glycerol per liter) supplemented by trace elements (1.5 mg of CaCl_2_.2H_2_O, 50.1 mg of FeCl_3_.6H_2_O, 60.3 mg of Na_2_-EDTA, 0.54 mg of ZnSO_4_.7H_2_O, 0.3 mg of MnSO_4_.H_2_O, 0.48 mg of CuSO_4_.5H_2_O, 0.54 mg of CoCl_2_.6H_2_O per liter) was also applied in induction studies with the same procedure. All of the experiments were repeated at least 3 times and mean values were used for comparison.

### Materials

D-mannitol was purchased from Serva Electrophoresis GmbH (Article No. 28410; Heidelberg, Germany) and D-glucitol (D-sorbitol; Cat. No. 85532) obtained from Sigma-Aldrich Chemie GmbH (Taufkirchen, Germany). Other chemicals were purchased from Merck (Darmstadt, Germany), Sigma-Aldrich (Taufkirchen, Germany) or Fluka (Buchs, Germany). DNA modifying enzymes were obtained from Roche Applied Science (Mannheim, Germany) or New England Biolabs GmbH (Frankfurt, Germany). Oligonucleotides were synthesized by Eurofins MWG Operons (Ebersberg, Gemany).

### DNA manipulation and transformation

Standard molecular techniques including *E. coli *transformation were carried out according to Sambrook et al. [33]. *B. subtilis *was naturally transformed using "Paris Method" [32,34]. PCRs were performed using Pfu DNA polymerase from Promega GmbH (Mannheim, Germany) on a MiniCycler from Biozym and the DNA constructs were sequenced by GATC Biotech (Konstanz, Germany). DNA preparation kits from Qiagen (Hilden, Germany) were applied for chromosomal DNA or plasmid extraction according to the manufactures' instruction. DNA sequencing reagents (AutoRead sequencing kit) were obtained from GE Healthcare (Munich, Germany).

### Construction of expression vectors

The oligonucleotides used in this study are listed in Table 2. Chromosomal DNA of *B. subtilis *168 was applied as the template for PCR reactions. The promoter region of the mannitol operon (*P_mtlA_*) was amplified using s5526/s5527 (plasmid pKAM1). Shortening of the 5'-end of the *P_mtlA _*region was performed by s6209/s5527 (pKAM9). For construction of pKAM12, oligonucleotides s6209/s6213 were applied to remove the wild type Shine-Dalgarno of *P_mtlA_*. Additionally, the untranslated region (UTR) of *P_mtlA _*in pKAM12 was further shortened by s6209/s6727 (pKAM44). Moreover, gradual shortening of *P_mtlA _*5' was performed by s6792/s6213 (pKAM48), s6726/s6213 (pKAM43), s6793/s6213 (pKAM49), s6829/s6213 (pKAM57), s6210/s6213 (pKAM59), and s6830/s6213 (pKAM58). Mutation of the base pairs between -35 box and MtlR binding site of *P_mtlA _*was carried out by s7091/s6213 (pKAM92), s7065/s6213 (pKAM84), and s6688/s6213 (pKAM27) oligonucleotides. Construction of pKAM45 was performed by fusion PCR. Primary PCRs s6209/s6728 and s6729/s6213 were followed by final PCR s6209/s6213 using primary PCR products as a template. Plasmid pKAM52 was also constructed in the same way using s6209/s6800 and s6801/s6213 PCR products as a template in the final PCR by s6209/s6213 oligonucleotides. Amplification of the promoter region of *mtlR *(*P_mtlR_*) was carried out using s5799/s6392 (pKAM18). All of the amplified promoter fragments were double digested by *Nhe*I and *Afl*II in order to be fused to *lacZ *as the reporter gene in pSUN279.2 [5], a derivative of pMTLBS72 [35]. Plasmid pMTLBS72 is a *B.subtilis/E.coli *shuttle vector containing a pBR322 origin of replication for *E. coli *and pBS72 origin of replication for *B. subtilis*. Replicon pBS72, which has been isolated from *B. subtilis*, is a stable theta replicating plasmid with low copy number in *B. subtilis *(6 copies per chromosome) [36].

**Table 2:** Oligonucleotides used in this study

Name	Sequence (5'→3')	Purpose
*Expression vectors*		
s5526	AAAAAAGCTAGCGGCTCCTGAAACCAGGAG	Amplification of *P_mtlA_*
s5527	AAAAACTTAAGATATAAACCCTCCCTGTTTTG	Amplification of *P_mtlA_*
s5799	AAGCTAGCTACGATATTCCATAAAAAGC	Amplification of *P_mtlR_*
s6209	AAAAAGCTAGCTTTTTATTTTTAAAAAATTGTCACAGTCA	Shortening upstream of *P_mtlA_*
s6210	AAAAAAGCTAGCTAAAAAATTGTCACAGTCATGTGC	Shortening upstream of *P_mtlA_*
s6213	AAAAAACTTAAGTAAGATACAAAAATATGTTCAGAGA	Shortening downstream of *P_mtlA_*
s6392	AAAAAACTTAAGAGCCAATCTTGATGTGCGG	Amplification of *P_mtlR_*
s6688	AAAAAAGCTAGCTTTTTATTTTTAAAAAATTGTCACAGTCATGTGCCAAAGTCCTGAAATCTTTCAA TTGTATAGGGACTG	Mutation between -35 and MtlR binding site
s6726	AAAAAAGCTAGCTATTTTTAAAAAATTGTCACAGT	Shortening upstream of *P_mtlA_*
s6727	AAAAAACTTAAGAGAGAATGATGCTTCCCTTTG	Shortening downstream of *P_mtlA_*
s6728	ATACAATTCTTTCTAAAGAGGACTTTGGCACATG	Mutation between -35 and MtlR binding site
s6729	CCTCTTTAGAAAGAATTGTATAGGGACTGTAAGCGT	Mutation between -35 and MtlR binding site
s6792	AAAAAAGCTAGCTTTATTTTTAAAAAATTGTCACA	Shortening upstream of *P_mtlA_*
s6793	AAAAAAGCTAGCTTTTTAAAAAATTGTCACAGTC	Shortening upstream of *P_mtlA_*
s6800	CGCTTACAGTCCCTATACAAAACTTAGTAAAGAGGACTTTGGCAC	Mutation between -35 and MtlR binding site
s6801	CCAAAGTCCTCTTTACTAAGTTTTGTATAGGGACTGTAAGCG	Mutation between -35 and MtlR binding site
s6829	AAAAAAGCTAGCTTTAAAAAATTGTCACAGTCAT	Shortening upstream of *P_mtlA_*
s6830	AAAAAAGCTAGCAAAAATTGTCACAGTCATGTG	Shortening upstream of *P_mtlA_*
s7065	AAAAAAGCTAGCTTTTTATTTTTAAAAAATTGTCACAGTCATGTGCCAAACAGGACTTTACTTTCAA TTGTATAGGG	Mutation between -35 and MtlR binding site
s7091	AAAAAAGCTAGCTTTTTATTTTTAAAAAATTGTCACAGTCATGTGGGTTTGTCCTCTTTACTTTCAA TTGTATA	Mutation between -35 and MtlR binding site
s7098	AAAAAAGCTAGCAGCTATTGTAACATAATCGGT	Fusion of P*_groE_*-(*cre*)-UTR*_PmtlR_*
s7189	CCTTAAAACGCTTACAGCAATTCTTATAATAAAGAATCTCC	Fusion of P*_groE_*-(*cre_PmtlA_*)-UTR*_PmtlR_*
s7190	TGCTGTAAGCGTTTTAAGGAAACCTCTCTATATCCTCTA	Fusion of P*_groE_*-(*cre_PmtlA _*)-UTR*_PmtlR_*
s7191	CCATAAAACGCTTTCAACAATTCTTATAATAAAGAATCTCC	Fusion of P*_groE_*-(*cre_PmtlR_*)-UTR*_PmtlR_*
s7192	TGTTGAAAGCGTTTTATGGAAACCTCTCTATATCCTCTA	Fusion of P*_groE_*-(*cre_PmtlR _*)-UTR*_PmtlR_*
s7193	CCTGGTAACGCTTTCAACAATTCTTATAATAAAGAATCTCC	Fusion of P*_groE_*-(*cre_acsA_*)-UTR*_PmtlR_*
s7194	TGTTGAAAGCGTTACCAGGAAACCTCTCTATATCCTCTA	Fusion of P*_groE_*-(*cre_acsA _*)-UTR*_PmtlR_*
s7195	CCTGTTCACGCTTTCAGCAATTCTTATAATAAAGAATCTCC	Fusion of P*_groE_*-(*cre_mtlA_*)-UTR*_PmtlR_*
s7196	TGCTGAAAGCGTGAACAGGAAACCTCTCTATATCCTCTA	Fusion of P*_groE_*-(*cre_mtlA _*)-UTR*_PmtlR_*
s7237	GTAGAGGATATAGAGAGGTTTCCCAATTCTTATAATAAAGAATCTCC	Fusion of P*_groE_*-UTR*_PmtlR_*
s7238	GGAGATTCTTTATTATAAGAATTGGGAAACCTCTCTATATCCTCTAC	Fusion of P*_groE_*-UTR*_PmtlR_*
		
*Integration vector*		
s5069	AAAAAAGAATTCGATATCAGATCTACGCGTTAACCCGGGC	Amplification of *ermC*
s5070	AAAAAACAATTGAATCGATTCACAAAAAATAGG	Amplification of *ermC*
s5621	AAAAAAGGCGCCTGGATTACCGTCTCATCG	Amplification of *glcT*
s5622	AAAAAAGGATCCAACCGCTTCCGCCTCATGAA	Amplification of *glcT*
s5623	GTGTTAGTACGCCGTGCTT	Amplification of *ptsHI*
s5624	GTCGCAATCATAGGGAACAT	Amplification of *ptsHI*
s5809	AAAAAAGATATCAACGCCCTTGCCCTTTC	Amplification of *ycsN*
s5810	AAAAAAAGATCTGCATCAGCTGGTAAACTGAT	Amplification of *ycsN*
s5812	AAAAAAAGGCCTAACACAAATGTTGTTTCTGC	Amplification of *ydaB*
s5860	AAAAAAACTAGTACCTGCATGGCACACGT	Amplification of *ydaB*
s5866	AAAAAAGGATCCATAAGAATTGACCTCCTCT	Amplification of *glcT-P_ptsGHI_*
s5867	AAAAAAGGATCCTAAGGGTGTTAGTACGCCGT	Amplification of *ptsHI*
s5918	AAAGATCTAACCAGGAGCCTTTTTATTTT	Amplification of *P_mtlA_*
s5919	CGAAATGTAAGGCGATCATATATAAACCCTCCCTGTT	Amplification of *P_mtlA_*
s5920	AACAGGGAGGGTTTATATATGATCGCCTTACATTTCG	Amplification of *mtlD*
s5921	AAGATATCGACCGTAAACAGCTTCCGTT	Amplification of *mtlD*
s5994	AAACTAGTAAGAAACTTAATCAATAACCGAC	Amplification of *ycsA*
s5995	AAAGGCCTTCTCGATTCCGCTATAATCAG	Amplification of *ycsA*
s6067	CCTGAAAGAAACACCATGCCCGAAC	Amplification of *ycnL*
s6068	AAGATATCGAAAGAAACACCATGCCCGAAC	Amplification of *ycnL*
s6079	AAAAAAACTAGTCTTTGGCACATGACTGTGACA	Amplification of *ycnL*
s6080	AAAGATCTCTTTGGCACATGACTGTGACA	Amplification of *ycnL*
s6302	AAAAAAGAATTCGGTATCTATCTTTTATGCCAA	Amplification of *ydjC*
s6303	AAAAAAGCTAGCTACGTAGTTCTGTCAGCAATC	Amplification of *ydjC*
s6304	AAAAAACTTAAGATCATTGAAGATGTTTCTTGA	Amplification of *pspA*
s6305	AAAAAACATATGCAGCAATTTGATTCGCCGC	Amplification of *pspA*
s6344	AAAAAAGATATCGATCGCCTTACATTTCGGTGC	Amplification of *mtlD*
s6345	AAAAAACATATGTTAAAATGATGGCGTGCAACG	Amplification of *mtlD*
s6865	GCTGACGGCCGGCTCCAGATCT GCA ATC AAG CCT TCA TAT AA	Amplification of *mtlR-H342D*
s6866	TTATATGAAGGCTTGATTGCAGATC TGG AGC CGG CCG TCA GC	Amplification of *mtlR-H342D*
s6867	AAAAAA ACTAGT TTA CAG TAT GTT TTT TTC TTT CAT	Amplification of *mtlR-H342D*
s6949	AAAAAA CCCGGG TAC GAT ATT CCA TAA AAA GC	Amplification of *mtlR-H342D*
*Primer extension*		
s5959	**Cy5**-GCTGCAAGGCGATTAAGTTGG	Hybridized to *lacZ*
s5960	**Cy5**-CCAGTCACGACGTTGTAAAAC	Hybridized to *lacZ*

Fusion of the *cre *sites of *P_mtlA_, P_mtlR_*, the internal *cre *site of *mtlA*, and *cre *site of *acsA *to P*_groE _*was performed by fusion PCR. In this way, the primary PCRs s7098/s7189 and s7190/s6392 (pKAM88), s7098/s7191 and s7192/s6392 (pKAM89), s7098/s7193 and s7194/s6392 (pKAM90), s7098/s7195 and s7196/s6392 (pKAM91) and s7098/s7237 and s7238/s6392 (pKAM101) were followed by final PCR s7098/s6392. The final products *P_groE_*-(*cre*)-UTR*_PmtlR _*were double digested by *Nhe*I/*Afl*II and ligated into pSUN279.2.

### Construction of *mtl *and *gut *mutant strains

Deletion of *mtlR, mtlAF*, and *mtl *operon was performed by homologous recombination, replacing the gene of interest by erythromycin resistance gene via double crossover. Integration vector pSUN338.3 [5] was used as the parental vector for construction of the integration vectors in this study. To delete *mtlR*, downstream flanking gene of *mtlR*, namely *ydaB*, was amplified by s5860/s5812 and cloned into the vector, digested by *Spe*I/*Stu*I. Into the resulting vector the upstream gene *ycsN *of *mtlR*, amplified with oligonucleotides s5809/s5810, was inserted between the *Eco*RV/*Bgl*II sites resulting in the cassette of *'ycsN*-*ermC*-*ydaB' *(pKAM4). Deletion of the *mtlAF *was performed by the fusion of the *P_mtlA _*and *mtlD*. Fusion PCR was carried out applying s5918/s5919 and s5920/s5921 for the primary reactions, and the final PCR fragment using s5918/s5921 was digested by *Bgl*II/*Eco*RV in order to clone the fragment upstream and opposite direction of erythromycin in pSUN338.3. Amplification of *ycnL*, the upstream flanking gene of *mtl *operon, by s6067/s6079 was performed and the product fragment was cloned between the *Stu*I/*Spe*I sites. The resulting plasmid named pKAM5 contained the *'ycnL-ermC-P_mtlA_*-*mtlD' *cassette. Disruption of the *mtlD *was performed by amplification of *mtlD*, utilizing s6344/s6345 and cloning the resulting fragment with *Eco*RV/*Nde*I digestion into pSUN338.3. Erythromycin gene was amplified by s5069/s5070 and blunt-cloned into the plasmid by disruption of *mtlD' *via *Hin*cII restriction site (plasmid pKAM14). Transcription of the erythromycin resistance gene was in opposite direction to the *mtl *operon transcription after integration into the chromosome by double crossover. Deletion of the *mtl *operon was performed through the construction of pKAM6. Amplification of the upstream gene, *ycnL*, was performed by s6068/s6080, followed by cloning between *Eco*RV/*Bgl*II restriction sites of pSUN338.3. Downstream flanking gene, *ycsA*, was amplified by s5994/s5995 and cloned via *Spe*I/*Stu*I sites. The final sequence *'ycnL*-*ermC*-*ycsA' *was used for integration into the chromosome. Deletion of the *gut *operon was done by replacing the complete operon of glucitol (Δ*gutRBPydjE::cat*) by a chloramphenicol resistance gene. For this purpose, plasmid pMW363.1 (a derivative of pSUN338.3 harboring chloramphenicol resistance) was used as the parental plasmid. Amplification of the upstream flanking gene of *gut *operon, namely *ydjC*, was performed by s6302/s6303, followed by cloning into *Eco*RI/*Nhe*I cut sites. As the downstream flanking gene, *pspA *was amplified by s6304/s6305 and cloned into the plasmid through *Afl*II*/Nde*I digestion. The resulting plasmid, named pKAM13, was used for the deletion of *gut *operon in *B. subtilis *3NA.

Prior to transformation of *B. subtilis *3NA with plasmids pKAM4, pKAM5, pKAM6, pKAM13, and pKAM14, the DNA was linearized by *Pac*I in order to prevent the single crossover integration. The transformants were selected on LB plates supplemented with erythromycin or chloramphenicol, depending on the plasmid. Additionally, the selected mutants were counter-selected on LB plates containing spectinomycin in order to remove the single crossover mutants. Finally, the deletion on the chromosome was confirmed by PCR using the relevant primers. All of the mutants were cultured in minimal medium harboring 1% of either mannitol or glucitol as the sole carbon source in order to confirm the disability of growth with mannitol.

### Markerless deletion of *ptsG *mutant

Construction of the Δ*ptsG *mutant was carried out in two steps leading to a markerless mutant. The first step included a disruption of *ptsG *and a 5' part of *ptsH *by the integration vector pMW312.2. This plasmid was a derivative of pSUN356.7 [5]. Using oligonucleotides s5621/s5622, the upstream region of *ptsG *(*glcT*) was amplified. The amplified fragment was cut by *Kas*I/*Bam*HI and ligated into pSUN356.7, which was digested by *Kas*I/*Bgl*II. The resulting vector was subsequently cut by *Pst*I. Next, the amplified fragment with s5623/s5624, harboring the downstream region of *ptsG *('*ptsHI*), was integrated. Thereby, the integration vector, pMW312.2, was constructed. Naturally competent *B. subtilis *3NA cells were transformed by pMW312.2 and selected on LB agar plates supplemented with erythromycin and 0.5% (w/v) glucitol. The growth of the resulting strain was checked on minimal medium agar plates containing 0.5% (w/v) mannose as the sole carbon source. The deleted mutant (named MW312) could not grow on the mannose minimal medium due to the incapability of producing HPr (encoded by *ptsH*) and enzyme I (encoded by *ptsI*). In the second step, a new integration vector was constructed, containing the intact *ptsG-ptsHI *promoter region fused to the intact *ptsHI *region. For this purpose, the upstream region, *glcT-P_ptsGHI _*was amplified by s5621/s5866 and the downstream sequence, *ptsHI *region, was amplified by s5867/s5624. The fragments were cut by *Kas*I/*Bam*HI and *Bam*HI/*Pst*I, respectively, and integrated via a 3-fragment-ligation into *Kas*I/*Pst*I cut pMW312.2. The resulting integration vector pMW373.3 was used for transformation of naturally competent MW312 cells. For the selection procedure, minimal mannose medium was applied and the mannose positive transformants were counter-selected for erythromycin sensitivity. Finally, the chromosomal DNA of Δ*ptsG *(strain MW373) was extracted and checked by the relevant primers.

### Markerless integration of *P_mtlR_-mtlR-H342D *into the chromosome

Mutation of the histidine residue of *mtlR *to aspartate was carried out using fusion PCR. In this way, primary PCRs by oligonucleotides s6949/s6865 and s6866/s6867 were followed by final PCR using s6949/s6867 and PCR products of primary PCRs as the template. The resulting fragment *P_mtlR_*-*mtlR-H342D *was digested by *Xma*I*/Spe*I and cloned into pHM31 via *Xma*I*/Nhe*I restriction sites (pKAM66). To integrate the *mtlR *allele into the chromosome of *B. subtilis *KM15 (Δ*mtlR*), an integration system based on histidine auxotrophy was applied [37]. Briefly, *B. subtilis *KM15 was transformed by pHM30 harboring truncated *hisI *and spectinomycin resistant genes. The transformants were selected on LB-spectinomycin agar (strain KM162). Afterwards, strain KM162 was transformed by pKAM66 harboring intact *hisI *and *P_mtlR_-mtlR-H342D *and the transformants were selected on Spizizen salts agar supplemented with 1% glycerol. The transformants were then validated on LB-spectinomycin agar and Spizizen salts agar with no citrate and supplemented by 0.5% mannitol as the sole carbon source. Finally, the integration was confirmed by PCR (strain KM163).

### *β*-galactosidase assay

The β-galactosidase activity was measured by using *o*-nitrophenyl-β-galactopyranoside (ONPG) as a substrate according to Miller assay [32,38] with the modification of Sun & Altenbuchner [5].

### Primer extension

Determination of the transcription start site (TSS) of *P_mtlA _*and *P_mtlR _*was performed by primer extension method. For this purpose, two Cy5 5'-labeled oligonucleotides, i.e. s5959 and s5960 hybridizing to nucleotides 69 to 89 and 31 to 51 downstream of *lacZ *start codon, were designed. *B. subtilis *3NA harboring pKAM1 containing *P_mtlA_*-*lacZ *and pKAM18 containing *P_mtlR_*-*lacZ *were cultivated in LB medium and induced at OD_600 _of 0.4 by 0.8% mannitol. Total RNA was extracted 1 h after addition of mannitol at 37^°^C and 200 rpm using the Qiagen RNeasy mini kit (Hilden, Germany). Avian myeloblastosis virus reverse transcriptase and T7 polymerase (Roche, Mannheim, Germany) were applied for reverse transcription and DNA sequencing, respectively. The generated cDNAs and sequencing fragments were analyzed on a denaturing polyacrylamide sequencing gel (GE Healthcare).

## Results

### *B. subtilis P_mtlA _*activity

**Figure 2:**
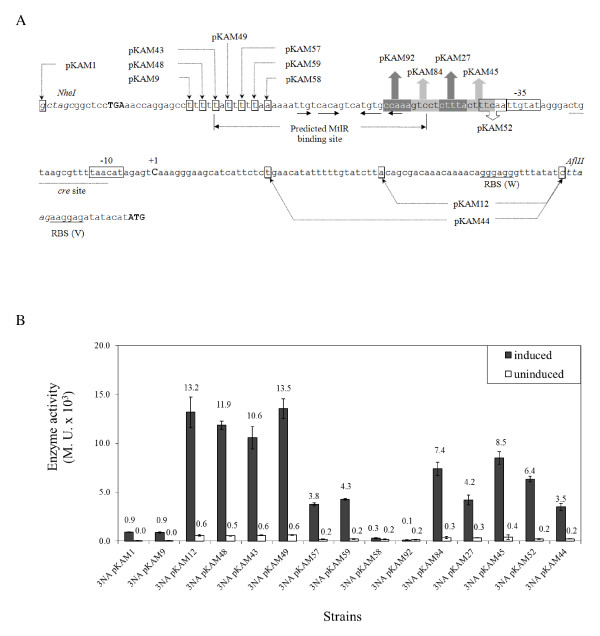
**Promoter of *mtl *operon fused to *lacZ *(A) and β-galactosidase activity of the constructs (B)**. (A) Upstream sequence of *lacZ *in plasmid pKAM1 and deletion derivatives are shown. The intervening sequence between stop codon of *ycnL *(bold capital letter) and *mtlA *start codon was obtained from *B. subtilis *and was inserted between *Nhe*I and *Afl*II site of the vector. It includes the promoter elements of *mtl *operon (*P_mtlA_*). The promoter -10 and -35 putative boxes are enclosed by rectangles, while the single C residue (bold capital letter) is the transcription start site. The determined *cre *site (underline) has an overlap with the -10 box. Ribosomal binding sites of the promoter (W) and vector (V) are underlined, and the *lacZ *start codon is marked in bold capital letters. The first bases of the shortened promoters are shown by boxes and the arrows. (B) Activity of different constructs of *P_mtlA _*in *B. subtilis *3NA containing the wild type promoter (pKAM1), the 5' shortened *P_mtlA_, i.e*., pKAM9, pKAM43, pKAM48, pKAM49, pKAM57, pKAM58, and pKAM59, as well as 3' shortened *P_mtlA_, i.e*. pKAM12 and pKAM44, were induced by 0.2% of mannitol at OD_600 _of 0.4. β-galactosidase activity of the cells was measured after 1 h of induction. Plasmids pKAM27, pKAM45, pKAM52, pKAM84, and pKAM92 contain the complementary base pairs in comparison to a wild type promoter in the discriminated region between the reported MtlR binding site and -35 box.

To characterize the promoter of the *mtl *operon (*P_mtlA_*), the complete sequence from the *ycnL *stop codon upstream of the *mtlA *promoter down to the start codon of *mtlA *was amplified by PCR and transcriptionally fused to the *lacZ *reporter gene in the *E. coli*/*B. subtilis *shuttle vector pSUN279.2 (Figure 2A). With this new plasmid pKAM1, competent *B. subtilis *3NA cells were transformed. The resulting strain was cultivated in LB broth and the *mtlA *promoter induced by adding 0.2% mannitol. In this way, the highest β-galactosidase activity was observed about one hour after the addition of mannitol (data not shown). Therefore, all further β-galactosidase activity measurements were performed at that time. The addition of the inducer mannitol resulted in a 21-fold induction, from 44 to 926 Miller units (M.U.).

The *mtl *operon is activated by MtlR. So far, the regulator binding site, -10 and -35 boxes of the *P_mtlA _*were reported by alignment of the *mtlA *promoter regions from *Geobacillus stearothermophilus *and *B. subtilis *[28]. Hence, shortening of the promoter region as used in pKAM1 was carried out for the determination of the boundaries of *P_mtlA_*. In the first step, a deletion from the 5'-end of the promoter was performed removing 20 bps. Determining β-galactosidase activity of this construct, namely pKAM9, showed that this deletion had no effect on the promoter activity (Figure 2B). The deleted sequence embraced the transcription terminator region of the *ycnL *gene. Afterwards, pKAM9 was used to delete 31 bps from the 3'-end of *P_mtlA _*containing the *mtlA *wild type ribosomal binding site. Induction studies with this newly shortened plasmid (pKAM12) by mannitol showed a remarkable increase of β-galactosidase activity (about 13 to 15-fold) in the basal expression level, as well as under fully induced conditions, compared to pKAM1. This increase might be either due to the shortened untranslated region of mRNA or the presence of two ribosomal binding sites in pKAM1, one from the vector pSUN279.1 and one from *mtlA*. The latter one is deleted in pKAM12 as depicted in Figure 2A. Further deletion of the next 20 bps from the 3'-end reduced the activity drastically (pKAM44). Therefore, starting from pKAM12, a series of 6 deletions were made at the 5'-end of *P_mtlA _*to define the MtlR binding site more precisely. Each construct had two bps more deleted at the 5'-end, giving the plasmids pKAM48, pKAM43, pKAM49, pKAM57, pKAM59, and pKAM58. Deletion of the first two bps of the proposed MtlR binding site (pKAM49) had almost no influence on promoter activity. By deletion of the next 2 bps (pKAM57) and 4 bps (pKAM59), the promoter was still inducible, but showed about a 3-fold reduced activity. Finally, deletion of the next two bps (pKAM58) had a drastic effect and led to a complete loss of promoter activity (Figure 2B). To define the 3'-end of the MtlR binding site, the region between the -35 promoter sequence and the proposed MtlR binding site was exchanged by a complementary DNA sequence. This was done in steps of 5 bps each leading to the plasmids pKAM52, pKAM45, pKAM27, and pKAM84. In all plasmids the promoter was still inducible but β-galactosidase activity was reduced. Finally, exchange of the 5 bps in the palindromic sequence of MtlR binding site in pKAM92 inactivated the *P_mtlA_*.

### Studying *P_mtlR _*activity

**Figure 3:**
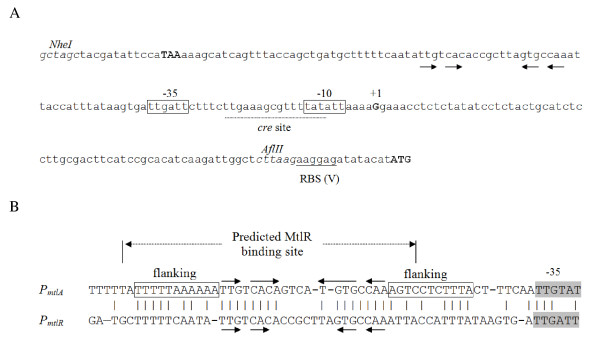
**Promoter of *mtlR *fused to *lacZ *(A) and alignment of *P_mtlA _*and *P_mtlR _*operators (B)**. (A) Upstream sequence of *lacZ *in pKAM18. The promoter region of *mtlR *locating downstream of *ycsN *stop codon (bold capital letter) and 8 bps upstream of the *mtlR *start codon is placed between *Nhe*I and *Afl*II sites of the vector pSUN279.2. The -10 and -35 boxes (rectangles), as well as transcription start site (G residue; bold capital letter), and *lacZ *start codon (bold capital letters) are shown. The *cre *sequence overlaps the Pribnow box (underlined). (B) Alignment of the DNA sequences 55 bps upstream of -35 boxes of *P_mtlA _*and *P_mtlR _*including the MtlR binding sites.

The promoter region of *mtlR*, amplified by PCR, included the stop codon of the gene *ycsN *upstream of *mtlR *and ended at 15 bps upstream of *mtlR *start codon (Figure 3A). The fragment without the putative *mtlR *ribosomal binding site was placed in front of the *lacZ *in pSUN279.1 (pKAM18). Subsequently, *B. subtilis *3NA was transformed by pKAM18 in order to monitor the *P_mtlR _*activity. Similar to *P_mtlA_*, mannitol was able to induce *P_mtlR_*. However, *P_mtlR _*showed a remarkably lower activity. The β-galactosidase activity in the non-induced cells was 23 M.U. and was raised 4.1-fold to 95 M.U. in 1 h (Table 3). Obviously, MtlR is able to autoinduce its own synthesis. When MtlR is able to induce its own synthesis, there should be a MtlR binding site upstream of *P_mtlR _*similar to *P_mtlA_*. Indeed, when the upstream sequences of both promoters were aligned, a 42 bps sequence was identified at a distance of 7 bps to the -35 promoter sequences, which share high sequence identity (Figure 3B).

**Table 3:** Activity of *P_mtlA _*(pKAM12) and *P_mtlR _*(pKAM18) in *B. subtilis *3NA and mutants thereof

	*B. subtilis*								
	
Treatment (0.2%)	3NA pKAM12	KM12 pKAM12	KM15 pKAM12	KM37 pKAM12	KM163 pKAM12	3NA pKAM18	KM12 pKAM18	KM15 pKAM18	KM37 pKAM18
Mannitol	13178 ± 1563	13862 ± 1342	92 ± 49	10801 ± 3290	13722 ± 1231	95 ± 25	243 ± 34	16 ± 1	156 ± 39
Mannitol + Glucose	5920 ± 894	10289 ± 1131	61 ± 46	1921 ± 967	11795 ± 729	28 ± 18	143 ± 14	5 ± 1	53 ± 21
Glucose	482 ± 86	10493 ± 1569	57 ± 42	1021 ± 379	6208 ± 583	14 ± 4	135 ± 39	5 ± 1	24 ± 4
Mannitol + Xylose	13693 ± 1343	14084 ± 2081	81 ± 45	11762 ± 3814	12499 ± 2112	99 ± 30	214 ± 33	15 ± 2	148 ± 49
Xylose	555 ± 120	14046 ± 2391	84 ± 52	2834 ± 943	1879 ± 76	25 ± 3	217 ± 41	15 ± 1	46 ± 7
Glucitol	8009 ± 947	12519 ± 1012	81 ± 53	11841 ± 3728	12425 ± 3297	51 ± 26	192 ± 43	10 ± 1	143 ± 42
Glucitol + Glucose	4559 ± 555	10743 ± 663	65 ± 51	1492 ± 611	11555 ± 2364	26 ± 22	142 ± 36	6 ± 1	44 ± 13
Uninduced	594 ± 83	15056 ± 2420	95 ± 59	2838 ± 933	2389 ± 645	23 ± 4	265 ± 71	16 ± 1	47 ± 10

The strains were induced by 0.2% (w/v) of sugars, or no inducer was added (control). β-galactosidase activity was measured 1 h after addition of inducer. Activity was calculated as Miller units. Mutations: KM12 (Δ*mtlAF*); KM13 (Δ*mtlAFD*); KM15 (Δ*mtlR*); KM37 (*mtlD::ermC*); KM163 (*mtlR-H342D*).

### Determination of the transcriptional start sites of *P_mtlA _*and *P_mtlR_*

**Figure 4:**
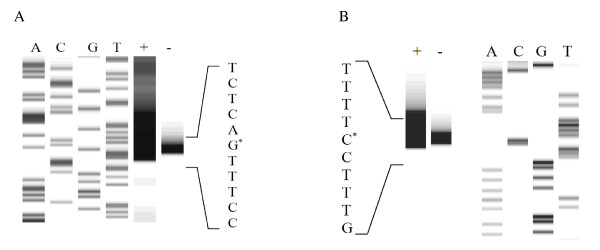
**Primer extension**. (A) Primer extension of *P_mtlA _*in pKAM12. The procedure has been explained in Methods. A, C, G, and T represent the dideoxynucleotide triphosphates used for the pKAM1 sequencing, and the + and - display the primer extension reaction of induced and uninduced sample, respectively. (B) Primer extension of *P_mtlR _*in pKAM18 and DNA sequencing reaction.

In order to determine the transcription start sites (TSS) of *P_mtlA _*and *P_mtlR_*, primer extension experiments were performed. *B. subtilis *3NA containing the plasmid pKAM1 and pKAM18, respectively, was cultivated in LB liquid medium and RNA was extracted from cells induced by mannitol and from non-induced cells. For designing of the Cy5-5'-labeled primers, the sequence of *lacZ*, which is identical in the two vectors, was chosen. Using primer extension technique, TSS of the *P_mtlA _*was identified at a C residue locating 92 bps upstream of the *lacZ *start codon, namely 72 bps upstream of *mtlA *(Figure 4A). Accordingly, -10 (TAACAT) and -35 (TTGTAT) boxes were determined resembling the house-keeping sigma factor (σ^A^) binding site. Besides, one *cre *site (CTGTAAGCGTTTTAA) within the sequence of the *P_mtlA _*was found having 2 mismatches (underlined) compared to the consensus sequence (WTGNAARCGNWWWCA) (Figure 2A) [39]. The *cre *site has an overlap with the -10 box of *P_mtlA_*. Likewise, the transcription start site of *P_mtlR _*was identified to be a single G residue (Figure 4B). Accordingly, -10 box (TATATT), -35 box (TTGATT), along with *cre *site (TTGAAAGCGTTTTAT), were determined (Figure 3A). Also in this case, the *cre *site overlaps with the Pribnow box and has 2 mismatches compared to the *cre *consensus sequence (underlined).

### Induction and catabolite repression of *P_mtlA _*and *P_mtlR_*

**Figure 5:**
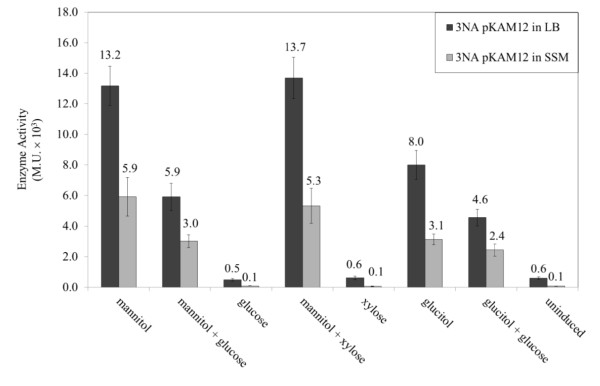
**β-galactosidase activity of *B. subtilis *3NA pKAM12**. β-galactosidase activity of *B. subtilis *3NA pKAM12 induced in LB medium compared to Spizizen salts medium (SSM) 1 h after addition of inducer.

Induction and catabolite repression of *P_mtlA _*and *P_mtlR _*on the plasmids pKAM12 and pKAM18 in *B. subtilis *3NA was investigated by cultivating the strains in LB liquid medium, as well as in Spizizen salts medium, with the addition of either mannitol, glucose, the non-PTS sugars glucitol and xylose, alone or in combination of two sugars (Figure 5). After one hour of induction, promoter activity was monitored by β-galactosidase activity. Although the promoter activity was almost doubled in LB medium, the *P_mtlA _*was less inducible in LB compared to SSM caused by a higher basal activity. However, comparison of the minimal and rich medium showed no significant difference in the glucose repression in *P_mtlA _*(ratio mannitol/mannitol + glucose). Therefore, further measurements were performed in LB medium due to a higher activity of the promoter and a shorter lag phase of growth. As shown in Table 3, both promoters were induced by mannitol and to a lesser extent by glucitol, whereas xylose had no effect. The presence of glucose repressed promoter activity during induction by mannitol and glucitol; as a result, *P_mtlA _*activity dropped at least twofold and *P_mtlR _*activity about threefold. This means that both promoters underlie catabolite repression.

### Deletion of *mtl *and *gut *genes

**Figure 6:**
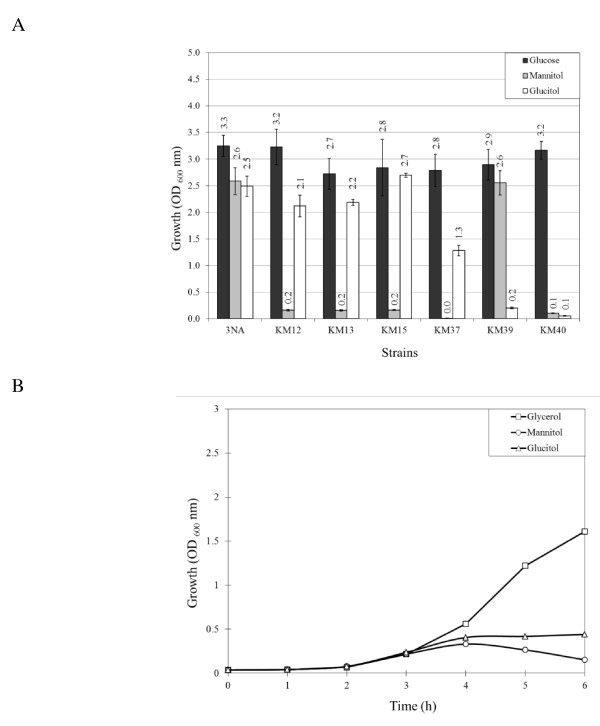
**Growth of *mtl *and *gut *mutants (A) and growth curve of *mtlD::ermC *mutant (B)**. (A) Growth of the *B. subtilis mtl *and *gut *mutants in minimal medium. Optical density at 600 nm of the strains KM12 (Δ*mtlAF*), KM13 (Δ*mtlAFD*), KM15 (Δ*mtlR*), KM37 (*mtlD::ermC*), KM39 (Δ*gutRBPydjE*), KM40 (Δ*gutRBPydjE *Δ*mtlAFD*), as well as of *B. subtilis *3NA (wild type), was measured after 16 h incubation at 37^°^C on a rotary shaker with 200 rpm. Minimal medium was supplemented by 1% of either mannitol, glucitol or glucose (control) as the sole carbon source. (B) The toxicity effect of the mannitol and glucitol in KM37. Strain KM37 was grown in LB medium containing 1% glycerol, mannitol, or glucitol and the growth curve was monitored at intervals of 1 h.

Genes involved in mannitol utilization, i.e. *mtlA, mtlF, mtlD*, and *mtlR*, were replaced or disrupted by an erythromycin resistance gene in order to investigate their probable influence on *P_mtlA _*activity, as well as on cell growth, with mannitol or glucitol as carbon source. The mutants, i.e. KM12 (Δ*mtlAF::ermC*), KM13 (Δ*mtlAFD::ermC*), KM15 (Δ*mtlR::ermC*) and KM37 (*mtlD::ermC*) were used to inoculate minimal medium with 1% mannitol to an optical density of 0.01 (OD_600_) and incubated for 16 h. Very weak growth of bacteria to an OD_600 _of maximum 0.2 was detected with KM13, KM12, and KM15 strains; however, KM37 strictly showed no growth (Figure 6A). Additionally, the *mtl *mutants were cultured in minimal medium with 1% glucitol as the sole carbon source. The mutants KM12, KM13, and KM15 grew similar to the wild type. However, KM37 showed half of the growth of the wild type. This phenomenon was reported by Watanabe et al. [28] at first.

As described, KM37 was the only strain that strictly showed no growth in the presence of mannitol as the sole carbon source and a reduced growth with glucitol. Consequently, in another effort the growth of KM37 in LB medium enriched by either 1% glycerol (as control), mannitol or glucitol was monitored. As depicted in Figure 6B, tracking the growth curve of KM37 at 1-h intervals revealed that the presence of mannitol and glucitol retarded the growth of the cells in the exponential phase. In fact, the maximal cell density observed with KM37 was 0.3 OD_600 _with mannitol and 0.4 OD_600 _with glucitol after 6 h incubation. By microscopy, we discovered that the cells became very swollen during incubation probably due to the accumulation of mannitol 1-phosphate. The same phenomenon was observed in the presence of glucitol, where probably glucitol 6-P is formed.

Besides the *mtl *operon, the complete *gut *operon, namely *gutRBPydjE*, was replaced by the chloramphenicol resistance gene in order to investigate the probable interaction of the two degradation pathways. The *gut *genes were deleted in *B. subtilis *3NA as well as in KM13 (Δ*mtlAFD*::*ermC*). The latter strain was used as the control in which no growth should be observed for both sugars. Growth of KM39 (Δ*gutRBPydjE::cat*) in minimal medium containing either 1% glucitol or 1% mannitol as the sole carbon source was strongly reduced in the case of glucitol, whereas for mannitol it was not affected. In the double mutant KM40 growth on either glucitol or mannitol was strongly reduced as expected (Figure 6A).

### Activity of *P_mtlA _*and *P_mtlR _*in *mtl *mutants

The activity of *P_mtlA _*in the absence of mannitol PTS components was investigated to determine if these components had an effect on the promoter activity. The strain KM12 lacks *mtlA *and *mtlF *encoding the EIICB^Mtl ^and EIIA^Mtl^, respectively. Strain KM12 was transformed by pKAM12 and β-galactosidase activities were determined in the presence or absence of mannitol, glucitol, or xylose of the resulting strain. The results indicated that the activity of *P_mtlA _*was constitutive (Table 3). In other words, the promoter showed highest activity regardless of the presence or absence of the inducer. Obviously, the components of mannitol transporter had an inhibitory effect on the *P_mtlA _*activity in the absence of mannitol. However, glucose repression was functional to some extent, e.g. 1.3-fold reduction of β-galactosidase activity by mannitol and 1.2-fold by glucitol when glucose was added.

Next, the *mtlD *deficient mutant KM37 was transformed by pKAM12. The deficiency of the mannitol 1-phosphate dehydrogenase not only increased the basal expression level of *P_mtlA _*(4.7-fold), but also enhanced the glucose repression from 2.2-fold in the wild type to 5.6-fold in the mutant. As expected, addition of mannitol or glucitol inhibited the growth during the 1 h of induction. However, the cells grew normally when glucose was added (data not shown). In accordance with pKAM12, similar results were obtained with pKAM18 in KM12 and KM37 mutants (Table 3). Surprisingly, *P_mtlA _*was equally induced by glucitol and mannitol (approx. 11,000 M.U.). Finally, deletion of the *mtlR *encoding the activator of the operon drastically decreased the activity of the *P_mtlA _*to about 95 M.U. (KM15 pKAM12, Table 3). This value is six times lower compared to the uninduced promoter in a wild type strain. No remarkable difference was observed between the different sugars tested, although addition of glucose reduced the activity slightly (1.2 to 1.5- fold). Finally, KM15 (Δ*mtlR::ermC*) was transformed by pKAM18 harboring *P_mtlR_*. In this mutant the promoter was no longer inducible. This confirms the assumption that *P_mtlR _*is an autoregulatory promoter. The maximum activity was reduced to 16 M.U., which was approximately the basal activity of *P_mtlR _*in the wild type strain, and the presence of glucose led to a further reduction.

### Phosphorylation of MtlR by HPr (His15~P)

It is known that PEP-dependent phosphorylation of HPr (His15) plays an essential role in the activity of PRD containing regulators such as ManR [5] and LevR [40]. Therefore, strain TQ432 harboring *ptsH-H15A *mutation was transformed by pKAM12. Results of induction of the TQ432 pKAM12 by different sugars are shown in Table 4. Similar to Δ*mtlR *mutant, no induction was observed by mannitol or glucitol. Moreover, no glucose repression was observed on the basal expression level due to a slow metabolism of the glucose. These results were in line with the *in vitro *studies of Joyet et al. [29] in which phosphorylation of PRDII domain of MtlR was observed in the presence of HPr (H15~^32^P).

**Table 4:** Activity of *P_mtlA _*(pKAM12) and *P_mtlR _*(pKAM18) in *B. subtilis *3NA, and CCR mutants

	*B. subtilis*						
	
Treatment (0.2%)	3NA pKAM12	TQ303 pKAM12	MW373 pKAM12	TQ432 pKAM12	3NA pKAM18	TQ303 pKAM18	MW373 pKAM18
Mannitol	13178 ± 1563	6120 ± 1701	11301 ± 628	61 ± 7	83 ± 3	93 ± 4	77 ± 17
Mannitol + Glucose	5920 ± 894	1636 ± 437	11788 ± 550	56 ± 7	19 ± 1	38 ± 3	73 ± 17
Glucose	482 ± 86	287 ± 158	398 ± 137	58 ± 8	12 ± 2	29 ± 1	25 ± 5
Mannitol + Xylose	13693 ± 1343	4982 ± 628	10533 ± 712	51 ± 7	84 ± 8	86 ± 2	74 ± 16
Xylose	555 ± 120	387 ± 32	417 ± 54	44 ± 9	25 ± 3	33 ± 3	25 ± 5
Glucitol	8009 ± 947	3489 ± 854	5211 ± 720	43 ± 5	40 ± 11	68 ± 4	41 ± 9
Glucitol + Glucose	4559 ± 555	774 ± 255	5324 ± 432	37 ± 3	17 ± 8	31 ± 2	40 ± 10
Uninduced	594 ± 83	501 ± 61	433 ± 115	59 ± 7	23 ± 4	33 ± 1	25 ± 6

Expression of *lacZ *by *P_mtlA _*(pKAM12) and *P_mtlR _*(pKAM18) in *B. subtilis *3NA as well as in CCR affected mutants, such as TQ303 (Δ*ccpA*), TQ432 (*ptsH-H15A*), and MW373 (Δ*ptsG*) were measured as described in the legend of Table 3.

### Activity of *P_mtlA _*and *P_mtlR _*in CCR deficient mutants

By transforming *B. subtilis *TQ303, which lacks the *ccpA *gene, a central element of the catabolite repression system, the effect of catabolite repression on *P_mtlA _*was investigated with plasmid pKAM12. This mutant grew slower than the wild type and showed a longer lag phase. In contrast to our expectations, the *ccpA *mutant demonstrated a similar or even slightly stronger catabolite repression than the wild type (3.7 versus 2.2-fold) when mannitol together with glucose were added (Table 4). The higher rate of repression might be caused by the fact that the β-galactosidase activity after 1 h induction with mannitol alone was about threefold lower than in the wild type, whereas the basal activity of *P_mtlA _*in the *ccpA *mutant resembled the wild type.

In addition to *P_mtlA_*, regulation of *P_mtlR _*was considered by the transformation of TQ303 mutant with pKAM18. The fully induced *P_mtlR _*by mannitol showed about the same activity as in the wild type in contrast to *P_mtlA_*. By adding of mannitol together with glucose, the β-galactosidase activity was less reduced compared to the wild type strain. On the other hand, the basal expression level in LB and LB with glucose or xylose was slightly increased at about the same amount as the catabolite repression was reduced (Table 4).

### Function of *cre *sites downstream of promoter

**Figure 7:**
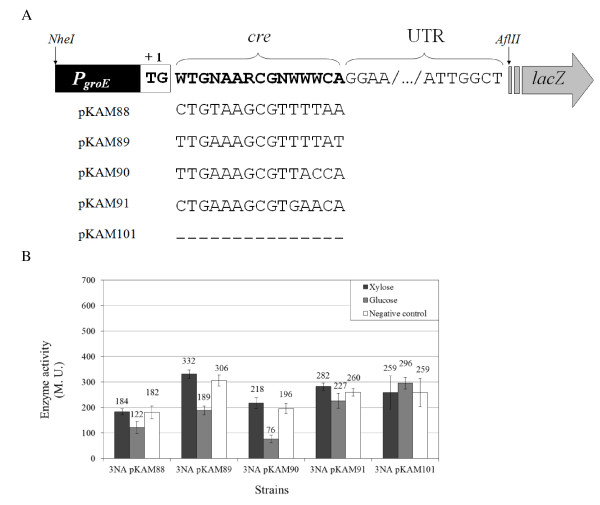
**Fusion of *cre *sites to P*_groE _*(A) and repression of the constructs by glucose (B)**. (A) Insertion of the *cre *sites of *P_mtlA _*(pKAM88), *P_mtlR _*(pKAM89), and of the internal *cre *site of *mtlA *(pKAM91), as well as the *cre *site of *acsA*, between *P_groE _*and *P_mtlR _*untranslated region. As negative control, *P_groE _*was directly fused to *P_mtlR _*untranslated region (pKAM101). (B) Catabolite repression of *B. subtilis *3NA pKAM88, pKAM89, pKAM90, pKAM91, and pKAM101 in the presence and absence of glucose (PTS) and xylose (non-PTS).

In order to clarify whether the *cre *sites of the *P_mtlA _*and *P_mtlR _*are functional, they were inserted downstream of the transcription start site of a constitutive promoter. Insertion of *cre *site at the +1 transcriptional start site inhibits the promoter clearance in a road block mechanism and RNA polymerase (RNAP) can be stalled when the HPr (S46~P)-CcpA binds to DNA [41]. In this case, the promoter of the *groESL *operon (P*_groE_*) was fused to an untranslated region of *P_mtlR _*(UTR*_PmtlR_*) (Figure 7A). In addition to the *cre *sites of *P_mtlA _*and *P_mtlR_*, another *cre *site, locating inside the *mtlA *gene (+1156 to +1169), has been reported [22]. Comparison of this *cre *site with the *cre *consensus sequence revealed only one mismatch (Figure 7A). As a positive control, the functional *cre *site of *acsA *(acetyl-CoA synthetase) was applied [42,43], while direct fusion of P*_groE_*-UTR*_PmtlR _*was used as the negative control. The constructs P*_groE_*-(*cre*)-UTR*_PmtlR _*were fused to *lacZ *in pSUN279.2. Transformation of *B. subtilis *3NA by pKAM88 (*cre_PmtlA_*), pKAM89 (*cre_PmtlR_*), pKAM90 (*cre_acsA_*), and pKAM91 (*cre_mtlA_*) was carried out. Afterwards, the transformants were grown in LB and at the OD_600 _of 0.4 either glucose (0.2%), xylose (0.2%), or no sugar was added. For all 4 promoters containing a putative *cre *site a reduction of β-galactosidase activity was observed by the addition of glucose. For the *cre *site inside the *mtlA *it was only 1.2-fold. The *cre *sites of *P_mtlA _*and *P_mtlR _*reduced the activity of *P_groE _*1.5- to 1.6-fold, whereas the *cre *site of *acsA *repressed the promoter expression 2.6-fold.

### Deletion of *ptsG *relieves glucose repression of *P_mtlA _*and *P_mtlR_*

As demonstrated before, the putative *cre *sequences had weak effects on the catabolite repression of *P_mtlA _*and *P_mtlR_*. Thus, we hypothesized that the catabolite repression in this system is mainly due to phosphorylation and dephosphorylation of one of the PRD domains of MtlR via HPr and maybe via the IICBA^Glc ^transporter. The *ptsG *encoding the transporter of glucose is the first gene of an operon, followed by *ptsH *and *ptsI *[26,44]. Disruption or deletion of *ptsG *by an antibiotic resistance gene may result in the loss of expression of *ptsH *and *ptsI *encoding the HPr and Enzyme I proteins, respectively. Therefore, a two-step method was developed. In the first step *ptsG *was replaced by an erythromycin resistance gene. These mutants were unable to grow on mannose and other PTS sugars. In the second step, the antibiotic resistance gene was replaced by the promoter *P_ptsGHI _*in which the selection was based on regaining the ability to grow on mannose. The resulting strain MW373 was transformed by pKAM12 and pKAM18 and incubated in the presence of different sugars (Table 4). MW373 harboring pKAM12 was still inducible by mannitol and glucitol, but showed identical β-galactosidase activity when glucose was simultaneously added. Similarly, deletion of the *ptsG *led to a loss of the glucose repression in *P_mtlR _*(Table 4).

### Catabolite repression through dephosphorylation of MtlR (H342D)

As shown, glucose repression was completely abolished in Δ*ptsG *mutant. The absence of EIICBA^Glc ^in the cell could affect the level of fructose 1,6-bisphosphate in the cell and finally the CcpA-dependent CCR. But, on the other hand the weakness of the *cre *sites to repress the promoter activity led us to hypothesize that the EIICBA^Glc ^interacts with other regulatory components of mannitol PTS. Lately, it is shown that the mutation of His 342 to Asp in PRDII domain of MtlR reduced the catabolite repression [29]. Therefore, it is assumed that the dephosphorylation of MtlR by glucose PTS transporter leads to a catabolite repression. In order to confirm this observation in our construct, integration of the gene encoding the mutant MtlR (H342D) into the genome of Δ*mtlR *strain was performed by the markerless integration system based on histidine auxotrophy (see Methods). Transformation of the *mtlR-H342D *strain, namely KM163, by pKAM12 was followed by induction studies. As shown in table 3, only a slight catabolite repression was observed in this mutant, although the basal activity was increased due to the mutation of the MtlR. Surprisingly, glucose as the repressor of the system increased the activity of the *P_mtlA_*. This phenomenon was also observed by other PTS sugars such as mannose and sucrose, although fructose showed no influence (data not shown).

## Discussion

In this study, regulation of the *mtl *operon and its activator was investigated by fusion of the promoter region of *mtlAFD *and *mtlR *to *lacZ*. Identification of transcription start sites revealed that *P_mtlA _*and *P_mtlR _*have the σ^A ^promoter structure. Shortening the upstream region of *P_mtlA _*showed that the two first base pairs of the predicted binding site by Watanabe et al. [28] have no effect on the *P_mtlA _*activity. In fact, these two nucleotides are not part of the highly conserved sequence found between *P_mtlR _*and *P_mtlA _*operator regions, and therefore might not belong to the binding site. Changing the base pairs between the predicted MtlR binding site and -35 showed a reduction in the activity of the promoter, although the inducibility of the promoter remained intact. Generally, transcriptional activators in their active conformation bind as homodimers or homomultimers to their operators [45,46]. Presumably, the mutations between operator and -35, as well as shortening the 5'-end of the MtlR binding site, reduce the binding affinity of the MtlR monomer to the affected operator site, while binding of the second monomer to the intact operator site stabilizes the activator-operon complex. Consequently, mutation in one of the distal or proximal flanking sequences of the operator changes the stability of RNAP-promoter complex mediated by the activator, and thereby affects the overall expression of the promoter [46]. This could explain the inducible but lower activity of the *P_mtlA _*with a shortened MtlR binding site. Furthermore, alignment of the operator region of *P_mtlA _*and *P_mtlR _*indicated that the short palindromic sequence in the operator is enclosed by conserved flanking sequences, consisting of 11 base pairs (Figure 3B). In fact, the MtlR binding site seems to be longer than the predicted sequence extending towards the -35 box. Previously, DNA footprint studies also indicated that the operator of the mannitol promoter in *G. stearothermophilus *extends near to -35 box [47]. Apparently, the distance between regulator and RNAP binding sites is shorter than the distance found in promoters regulated by class I type activators, which is -61 to -91 bps. Consequently, MtlR is likely a class II type activator in which the regulator makes a direct contact to domain 4 of σ^70 ^[48-53].

Activity of *P_mtlA _*and *P_mtlR _*was monitored in *mtl *mutants by the presence of mannitol as the inducer, glucitol as a nonspecific inducer, and glucose as the effector of carbon catabolite repression. In the *mtlR *mutant, both *P_mtlA _*and *P_mtlR _*activities were nearly abolished. In contrast to most prokaryotic transcriptional regulators, PRD containing activators do not bind effector molecules and activity is controlled by phosphorylation and dephosphorylation. Accordingly, expression of *P_mtlA _*and *P_mtlR _*was also abolished in a HPr (H15A) mutant. These results were in line with recent *in vitro *studies on MtlR indicating that phosphate transfer from HPr (H15) to PRDII of MtlR is essential for stimulation of MtlR activity. This is similar to other PTS transcriptional activators [29]. On the other hand, deletion of the mannitol-specific PTS genes (*mtlAF*) resulted in a constitutive activity of *P_mtlA _*and *P_mtlR _*indicating an inhibitory effect of the EIICB^Mtl ^and EIIA^Mtl ^on *P_mtlA _*and *P_mtlR_*. MtlR belongs to the LevR-type regulator. Four such transcription activators are known in *B. subtilis *(LevR, LicR, ManR, and MtlR) [16]. The inhibitory effect of the specific transporters on their activators has been shown already for EII^Lev ^[54,55], EII^Cel ^[56], and EII^Man ^[5]. MtlR phosphorylation studies by Joyet et al. [29] have also indicated that the phosphorylation of MtlR by mannitol transporter inactivates the regulator.

The replacement of *mtlD *with erythromycin resistance gene made the cells sensitive to glucitol as well as mannitol. So far, it is clear that *mtlD *not only plays a vital role in the consumption of mannitol, but also is necessary for assimilation of glucitol. Induction of *P_mtlA _*in the *gutRBPydjE *deleted mutant showed that the *gut *system components, especially GutR, have no effect on *P_mtlA _*activity (data not shown). Northern hybridization studies, as well as induction studies, proved the ability of glucitol to induce the *mtl *operon [28]. A weak transport of glucitol by the mannitol specific PTS-transporter in *B. subtilis *was observed by Chalumeau et al. [30]. This could be due to a relaxed specificity of the PTS transporters in which the transporter may transport more than one sugar. So far, it is observed that PtsG takes up sucrose and salicin in addition to glucose, and the β-glucoside permease (BglP) is capable of a weak uptake of glucose [57,58]. Thus, the inducibility of *P_mtlA _*and *P_mtlR _*by glucitol is likely due to the uptake of glucitol by mannitol transporter where EIICB^Mtl ^and EIIA^Mtl ^dephosphorylate the Cys419 and H599 residues of MtlR, and activates the regulator. Previously, the weak activity of mannitol 1-phosphate dehydrogenase with sorbitol 6-phosphate was detected by Horwitz and Kaplan [59]. It can be assumed that a small amount of glucitol in the growth media is not taken up by the glucitol transporter, but is phosphorylated to glucitol 6-phosphate by EIICBA^Mtl ^during transport. Afterwards, mannitol 1-phosphate dehydrogenase oxidizes glucitol 6-phosphate to fructose 6-phosphate. Therefore, deletion of the mannitol dehydrogenase should lead to an accumulation of the non-metabolized glucitol 6-phosphate and finally to the killing of the cell.

In addition to *mtl *deficient mutants, catabolite repression of *P_mtlA _*and *P_mtlR _*was investigated in CcpA-dependent CCR mutants. Expression of *P_mtlA _*in the Δ*ccpA *mutant was dramatically reduced, although the catabolite repression was increased. In fact, the Δ*ccpA *mutant grew slower than the wild type strain due to pleiotropic roles of CcpA in the nitrogen regulation, branched chain amino acid synthesis as well as tricarboxylic acid cycle [60,61]. In contrast, catabolite repression of *P_mtlR _*was reduced in the Δ*ccpA *strain; however, the *P_mtlR _*activity was not completely relieved from CCR. Obviously, no exact interpretation was obtained by investigation of general *trans *elements of CCR. Therefore, we focused on the *cis *element of CCR, which is the *cre *site of *P_mtlA _*and *P_mtlR_*. The influence of *cre *sites from *P_mtlA_, P_mtlR_, acsA*, and *mtlA *were compared by using the constitutive promoter of *groESL *operon. In the presence of glucose the *cre *sites of *P_mtlA _*and *P_mtlR _*significantly repressed the expression of *lacZ *from P*_groE_*, whereas the *cre *site located inside the *mtlA *gene had almost no effect. Therefore, it seems that two weak *cre *sites, located in the regulator and operon promoter work together to repress the mannitol genes, whereas in the similar *B. subtilis *mannose system, a strong *cre *site in the promoter of the regulatory gene (*P_manR_*) is the only *cis *element for CcpA-dependent CCR [5]. These results were in line with the previous attempts in *mtl *operon representing the CcpA-dependent CCR in mannitol PTS [62,63].

So far, it is shown that the glucose repression is abolished when *ptsG *was disrupted [64,65]. In order to see the probable effect of the glucose PTS transporter, *ptsG *was deleted from the genome in a way that *P_ptsGHI _*was fused to *ptsHI*. As expected, activity of the *P_mtlA _*and *P_mtlR _*in the Δ*ptsG *mutant was identical in the presence and absence of glucose. The reason why we did not see any effect of the *cre*/CcpA system in the presence of glucose can be explained by the fact that HPr kinase is only active in the presence of glycolytic intermediates. Due to the deletion of *ptsG *no glucose is taken up by the EIICBA^Glc^. From the second glucose transporter (GlcU) it is known that this gene (*glcU*) is expressed 3 h after sporulation under the control of σ^G ^[66,67]. Finally, expression of *glcP *(glucose/mannose:H^+ ^symporter), a third glucose permease depends on glucose 6-phosphate produced by glucose PTS [65,68]; therefore, reduction of fructose 1,6-bisphosphate pool in the cell eliminates the *cre*/CcpA dependent CCR. Since CcpA-dependent CCR was completely abolished only in Δ*ptsG *and *ptsH-H15A *mutants, CcpA dependent CCR might not play the main role in the mannitol regulation. The strong effect of glucose might be explained by a hierarchy in the affinity of HPr (H15~P) to EIICBA^Glc ^versus EIIA^Mtl ^and PRDII domain of MtlR. Alternatively, EIICBA^Glc ^might also inactivate MtlR directly by dephosphorylation of PRDII domain. Mutation of the histidine 342 to aspartate located in PRDII domain of MtlR supported the assumption of dephosphorylation of MtlR by glucose PTS components. In this mutant addition of PTS sugars such as glucose, mannose, and sucrose (except fructose) increased the activity of the promoter. This could be due to dephosphorylation of the EIIA and EIIB domains of MtlAF/or MtlR by the specific transporters of glucose, mannose, and sucrose: However, further experiments are necessary to clarify this effect. Overall, catabolite repression in *mtl *system is mainly functional at posttranslational level by PtsG and at the transcription initiation level by CcpA dependent CCR, which switch off the expression of mannitol uptake system.

## Conclusions

Characterization and optimization of the promoter region of *mtl *operon, as well as its regulator, resulted in construction of a highly inducible expression system in *B. subtilis *based on mannitol as a cheap inducer. Activity of *P_mtlA _*and *P_mtlR _*in several mutants revealed that this system is mainly regulated by the phosphorylation and dephosphorylation state of the specific regulator. This accelerates the further manipulation of the strain to enhance the expression system.

## Competing interests

The authors declare that they have no competing interests.

## Authors' contributions

KMH designed and performed the experiments and analyzed the data. MW constructed the *ptsG *mutant and wrote the corresponding section in Materials. JA supervised and coordinated the project. KMH wrote the paper which was later revised and corrected by JA. All authors approved the final version of the manuscript.
